# Supplementary material for: The myogenic electric organ of Sternopygus macrurus: a non-contractile tissue with a skeletal muscle transcriptome
Source: PeerJ. 2016 Apr 14;4:e1828. doi: 10.7717/peerj.1828 (PMC4841239; doi:10.7717/peerj.1828)
Supplement: Figure S1 — The following information is applied to all of the remaining figures—D. rerio KEGG pathway with colors representing expression levels of S. macrurus transcripts in EO vs. muscle. Names highlighted in yellow denote transcripts expressed at similar levels in EO relative to muscle. Names colored in light green represent genes mapped in D. rerio, but with no match annotated in S. macrurus. White boxes represent genes with no representative in D. rerio. [file peerj-04-1828-s001.pdf]

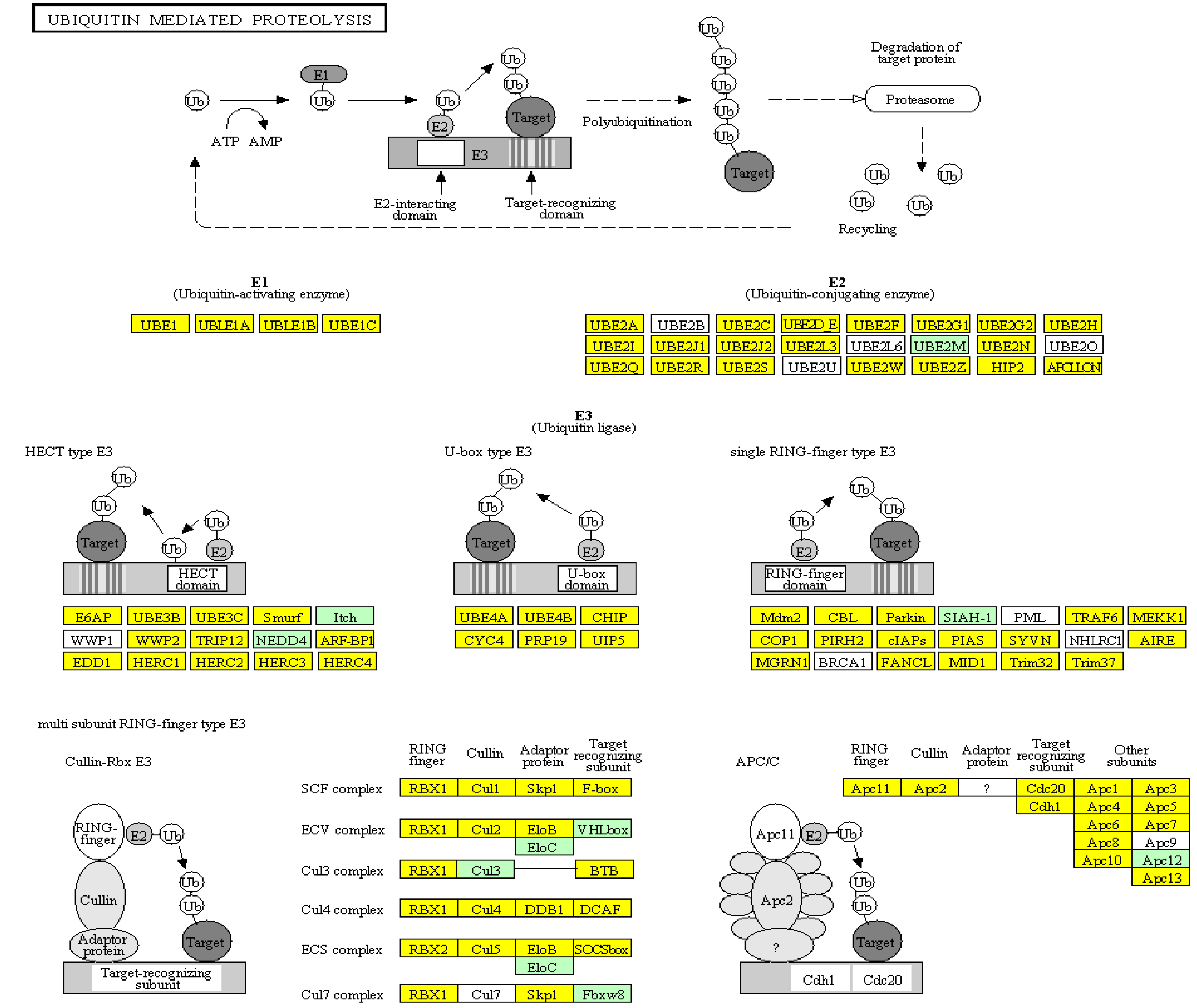

04120 6/8/12

(c) Kanehisa Laboratories
