# Supplementary material for: The myogenic electric organ of Sternopygus macrurus: a non-contractile tissue with a skeletal muscle transcriptome
Source: PeerJ. 2016 Apr 14;4:e1828. doi: 10.7717/peerj.1828 (PMC4841239; doi:10.7717/peerj.1828)
Supplement: Figure S2 — See caption of Fig. S1 for detailed description. [file peerj-04-1828-s002.pdf]

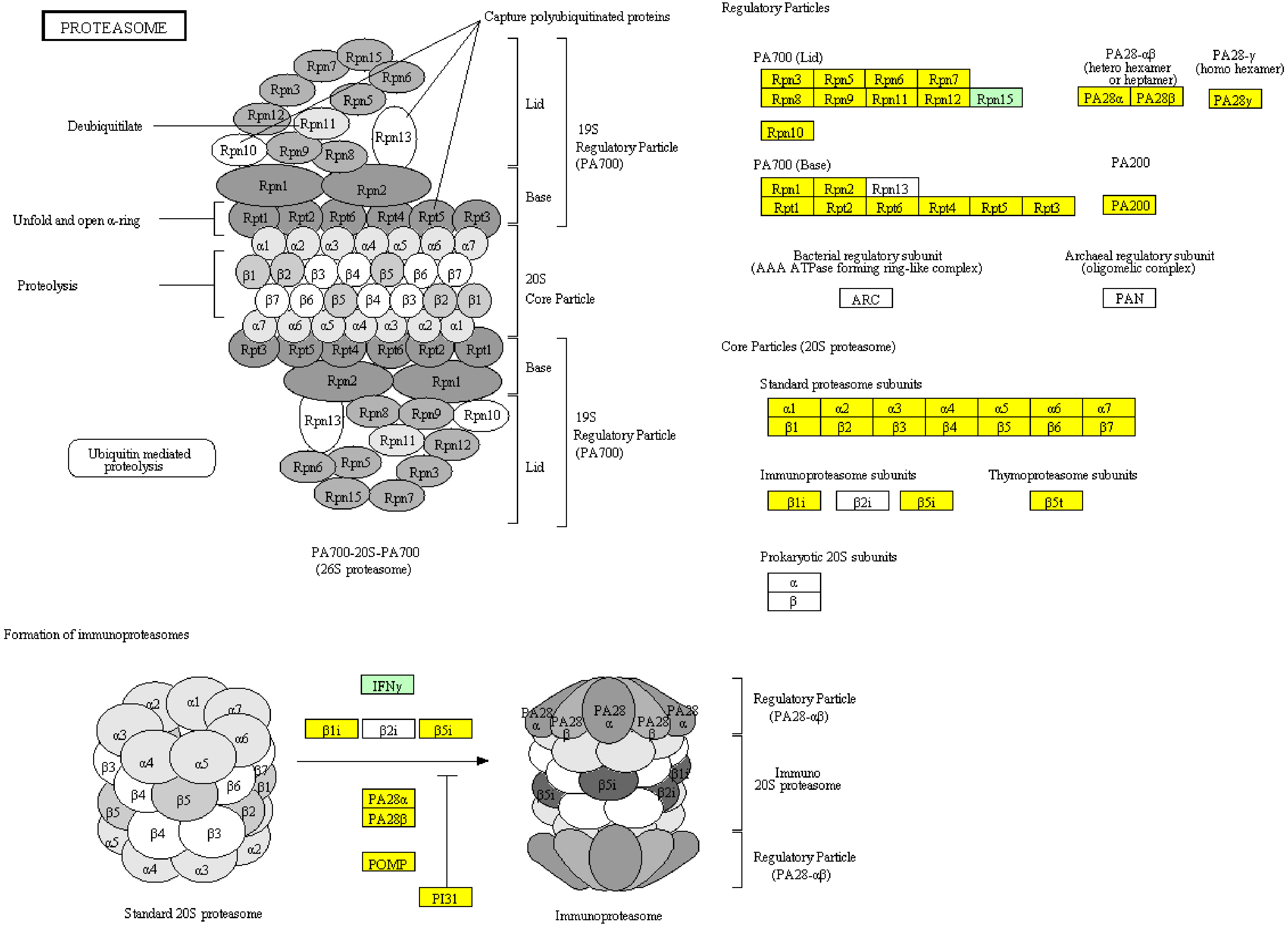

03050 12/1/10

(c) Kanehisa Laboratories
