# Supplementary figures and images for: The myogenic electric organ of Sternopygus macrurus: a non-contractile tissue with a skeletal muscle transcriptome
Source: PeerJ. 2016 Apr 14;4:e1828. doi: 10.7717/peerj.1828 (PMC4841239; doi:10.7717/peerj.1828)

# REGULATION OF AUTOPHAGY

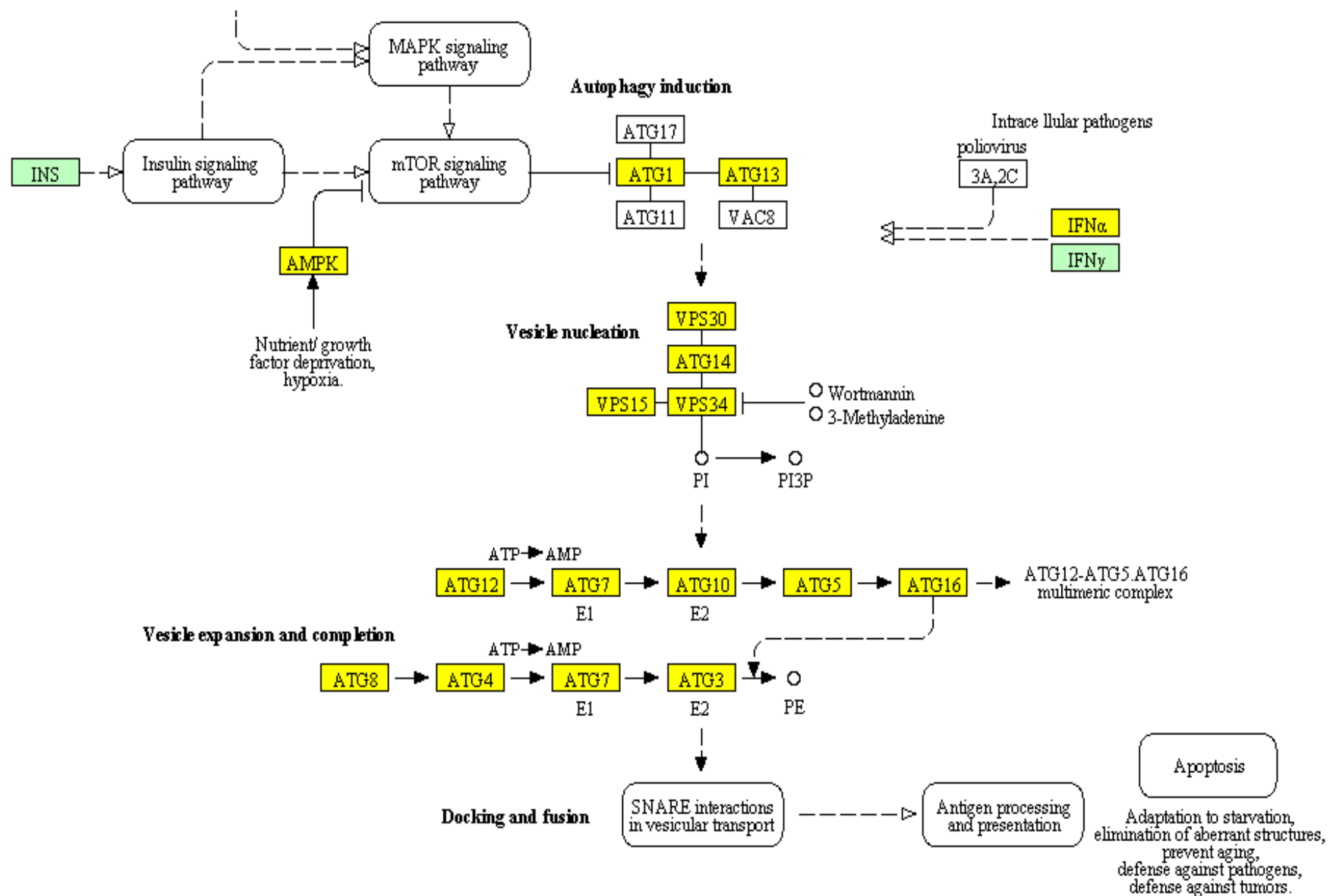

Supplement: Figure S3 — See caption of Fig. S1 for detailed description. [file peerj-04-1828-s003.pdf]
